# Instantaneous effects of mindfulness meditation on tennis return performance in elite junior athletes completing an implicitly sequenced serve return task

**DOI:** 10.3389/fspor.2022.907654

**Published:** 2022-08-23

**Authors:** Edward J. O'Connor, Alistair Murphy, Mark J. Kohler, Russell W. Chan, Maarten A. Immink

**Affiliations:** ^1^Allied Health and Human Performance, University of South Australia, Adelaide, SA, Australia; ^2^Alliance for Research in Exercise, Nutrition and Activity (ARENA), Adelaide, SA, Australia; ^3^Sports Science and Sports Medicine Unit, Tennis Australia, Melbourne, VIC, Australia; ^4^Adelaide Brain & Cognitive Development Laboratory, School of Psychology, University of Adelaide, Adelaide, SA, Australia; ^5^Cognition, Data and Education, University of Twente, Enschede, Netherlands; ^6^Sport, Health, Activity, Performance and Exercise (SHAPE) Research Centre, Flinders University, Adelaide, SA, Australia

**Keywords:** meditation, sport, performance, cognitive control, sequence learning, tennis, athlete, mindfulness

## Abstract

Single-session meditation augmentation of sport-specific skill performance was tested with elite junior tennis athletes. Athletes completed one of two styles of mindfulness meditation (focused-attention or open-monitoring) or a control listening condition prior to performing an implicitly sequenced tennis serve return task involving the goal of hitting a target area placed on the service court. Unbeknownst to athletes, six distinct serves followed a repeating second-order conditional sequence for two task blocks before the sequence was altered in a third transfer block. Task performance was operationalized as serve return outcome and analyzed using beta regression modeling. Models analyzed group by block differences in the proportion of returned serves (i.e., non-aces), returns placed in the service court, and target hits. Contrary to previous laboratory findings, results did not support meditation-related augmentation of performance and/or sequence learning. In fact, compared to control, meditation may have impaired performance improvements and acquisition of serve sequence information. It is possible that the effects of single-session meditation seen in laboratory research may not extend to more complex motor tasks, at least in highly-trained adolescents completing a well-learned skill. Further research is required to elucidate the participant, task, and meditation-related characteristics that might promote single-session meditation performance enhancement.

## Introduction

Mindfulness meditation is a form of mental training involving bouts of enhanced attention regulation to present-moment experiences with an attitude of acceptance and non-judgment (Kabat-Zinn, 1990). Mindfulness training has become an increasingly popular tool within elite sporting settings (Gross, 2020) due to evidence for meditation-related enhancement of a range of psychological, physiological, and cognitive factors underpinning performance (Pineau et al., 2014; Bühlmayer et al., 2017; Josefsson et al., 2019; Noetel et al., 2019; Corbally et al., 2020). Whilst most existing meditation research among athletes has investigated meditation-related performance benefits following prolonged periods of resource and time-intensive meditation training (Gross, 2020), an alternative line of research in non-athlete populations has begun to investigate whether attention and performance can be augmented through single bouts of meditation that immediately precede task completion (Leyland et al., 2019).

Though attention regulation is central to all contemporary descriptions of meditation (e.g., Bishop et al., 2004; Hölzel et al., 2011; Malinowski, 2013; Tang et al., 2015), techniques differ in *how* attention is regulated and thus the predominant neurocognitive mechanisms engaged during practice (Lutz et al., 2008; Lippelt et al., 2014). Lutz et al. (2008) proposed that techniques can be categorized as either focused attention meditation (FAM) or open-monitoring meditation (OMM). FAM involves narrow, selective attention to a single pursued object (e.g., physical sensations associated with respiration) to the exclusion of all other information. When distraction is noticed, FAM practitioners nonjudgmentally observe the distraction and return their focus to the pursued object (Lutz et al., 2008; Lippelt et al., 2014). As such, FAM engages cognitive control processes associated with maintenance of goal-relevant information, inhibition of task-irrelevant information, disengagement from distraction, and re-orienting of attention (Miyake et al., 2000; Chan et al., 2020). Conversely, OMM is characterized by a broad, flexible, and receptive state of attention during which participants are guided to maintain meta-cognitive awareness of their attention (Lutz et al., 2008). Instructions typically encourage participants to nonjudgmentally observe the contents of consciousness as it unfolds in the present-moment. These different styles exert divergent influence on cognitive control states, with the concentrative goal-oriented nature of FAM increasing cognitive control activation, whereas the expansive, receptive attentional state established in OMM weakens top-down cognitive control (Lippelt et al., 2014; Colzato and Hommel, 2017; Hommel and Colzato, 2017).

Interestingly, recent research has established that cognitive control states established in single bouts of meditation may endure to influence performance on subsequent cognitive tasks (Colzato et al., 2012, 2016; Mrazek et al., 2012; Lippelt et al., 2014; Chan et al., 2017, 2018, 2020; Immink et al., 2017; Zhu et al., 2020; Greif and Kaufman, 2021, though see for null results Baranski, 2021). For example, several studies have investigated the instantaneous effects of single-session meditation on performance using the Serial Reaction Time Task (SRTT; Nissen and Bullemer, 1987) paradigm (Chan et al., 2017, 2018, 2020; Immink et al., 2017). In each SRTT trial a stimulus appears at one of four locations horizontally arranged on the display. Participants respond to each stimulus by pressing a key corresponding to stimulus location. Unbeknownst to participants, stimuli are presented following a pre-specified pattern which repeats over a number of cycles within each learning block. Typically, several learning blocks are administered before the introduction of a transfer block featuring a different repeating sequence (for detailed descriptions, see Chan et al., 2017, 2020). Reaction time tends to shorten across SRTT learning blocks and this performance improvement can be derived through general practice effects, whereby repeated practice results in stronger stimulus-response mapping and thus expedited processing time for each individual trial (Hommel, 2000; Abrahamse et al., 2010). The resulting response strategy is deemed “stimulus-based responding” due to the reliance on features of each individual stimulus to signal the appropriate participant response (Immink et al., 2017). Stimulus-based responding is associated with increased cognitive control and is relatively resilient to alterations to the underlying sequence structure (Abrahamse et al., 2010; Chan et al., 2020). Additionally, performance gains across the SRTT can be achieved through improved plan-based responding (Chan et al., 2020). Here, internalization of the underlying sequence allows performance to become more anticipatory and thus less stimulus reliant. Participants who exhibit plan-based responding “chunk” several elements of the underlying sequence together (Jiménez, 2008) and rely on this internalized sequence representation to inform subsequent responses (Chan et al., 2020). As narrow focus to goal-relevant information and inhibition of other sources of data (i.e., increased cognitive control) impairs access to sequential information, plan-based responding is associated with reduced cognitive control (Borragan et al., 2016). Whilst plan-based responding yields effective performance in the context of the learned sequence, this response strategy is susceptible to significant performance detriments when the underlying sequence is altered in transfer blocks (Chan et al., 2017; Immink et al., 2017).

Recent evidence suggests that cognitive control states established in FAM and OMM may augment SRTT performance, with FAM promoting stimulus-based responding (Chan et al., 2017, 2018, 2020; Immink et al., 2017) and OMM resulting in greater plan-based responding though modulated by cognitive effort (Immink et al., 2017). For example, Immink et al. (2017) found that both FAM and OMM improved overall SRTT performance, as indexed by faster mean reaction time across the entire task, compared to a control condition. When FAM preceded the SRTT, performance benefitted from enhancement of stimulus-oriented responding. Conversely, OMM enhanced SRTT performance through greater sequence-oriented responding, particularly in those participants who perceived the OMM technique to be less effortful (Immink et al., 2017). This research suggests that meditation – *via* its capacity to modulate cognitive control states - may instantaneously influence sequential performance when deployed immediately prior to task performance. However, whether such instantaneous effects of meditation on performance are evident in applied settings, such as sport performance, remains unknown.

Execution of certain sport skills might be achieved through either proactive (i.e., plan-based) or reactive (i.e., stimulus-based) responding. For example, in tennis, it is well established that the server holds a significant advantage over the receiver in terms of point-winning probabilities (O'Donoghue and Brown, 2008; Gillet et al., 2009; Fitzpatrick et al., 2019). This advantage, however, can be diminished by enhancing the receiver's level of serve return performance (Gillet et al., 2009; Ma et al., 2013). To enhance their serve return performance, tennis athletes can utilize salient visual stimuli, for example, from the server's ball toss, as early information to inform serve trajectory and velocity (Vernon et al., 2018). In this situation, the athlete is relying on stimulus-based responding since the target response, the serve return, is determined by their use of a stimulus (e.g., the ball toss). The returner can even further diminish the server's advantage by using knowledge to anticipate an upcoming serve. This knowledge might represent serve patterns of the opponent dependent of situational factors such as the playing surface or match progression (Gillet et al., 2009; Vernon et al., 2018). Here, the anticipated response, the serve return, does not rely on a stimulus but rather on preestablished knowledge of a serve sequence. Accordingly, anticipatory action can be considered as sequence-based responding.

While research has established that visual attention, pattern recognition and anticipation skills are important determinants of serve return performance (Williams et al., 2002, 2004; Farrow and Reid, 2012; Loffing et al., 2015; Sáenz-Moncaleano et al., 2018), there is an absence of research that has addressed if the function of these skills can be enhanced by single-session meditation. Given that cognitive control can affect performance in both laboratory and sport tasks (McPherson and Vickers, 2004; Scharfen and Memmert, 2019), it is conceivable that the instantaneous effects of meditation states might enhance sport performance. That is, if previous laboratory findings (Chan et al., 2017, 2018, 2020; Immink et al., 2017) generalize to real-world sport skill scenarios, then a session of mindfulness meditation might modulate subsequent performance on a serve return task. Whether enhanced serve return performance arises from optimized stimulus-based responding or sequence-based responding, would depend on whether the preceding meditation involved a FAM or OMM technique, respectively (Immink et al., 2017). Sport skills such as returning a tennis serve, however, are more complex than the laboratory-based keyboard-pressing tasks given the former are performed in dynamic action environments and involve greater perceptual and motor demands. Moreover, whether meditation-based performance enhancement generalizes to youth athletes is not known since previous work with laboratory tasks has only involved adult participants (Chan et al., 2017, 2018; Immink et al., 2017). It is thought that cognitive control processes do not fully develop until early adulthood (Ferguson et al., 2021) meaning that younger athletes may not similarly benefit from single-session meditation as their adult counterparts. As a result, it may be that the instantaneous effects of mindfulness states on skilled performance, as observed in laboratory settings (Chan et al., 2017, 2018; Immink et al., 2017), do not elicit observable performance gains in applied sport settings with adolescent athletes.

The present study aimed to assess the instantaneous effects of FAM and OMM techniques on tennis serve return performance. In alignment with laboratory SRTT studies (Chan et al., 2017, 2018; Immink et al., 2017) it was hypothesized that both FAM and OMM would enhance serve return performance compared to an active control condition. As such, FAM and OMM groups were expected to achieve significantly higher proportion of successful returns across the task relative to control. Regarding distinct forms of sequential performance following FAM and OMM, performance after OMM was expected to reflect plan-based responding, where performance is significantly reliant on the learned sequence structure. In contrast, serve return performance following FAM was expected to reflect greater stimulus-based responding, whereby performance is maintained irrespective of the presence of an underlying sequence. Specifically, for the OMM group it was hypothesized that the proportion of successful returns would significantly diminish when the learned sequence was altered, whereas the FAM group would display consistent serve return odds across task blocks, regardless of any alteration to the underlying structure.

## Methods

### Participants

Thirty-three participants were recruited from Tennis Australia's National Youth Academy squads in Sydney and Adelaide. Three individuals were unable to participate due to sustaining injuries during match play prior to the data collection period, resulting in a total of 30 participants (15 females) with 16 participants based in Adelaide and 14 participants based in Sydney. Participant ages ranged from 13.6 to 19.1 years (*M*age = 16.34, *SD* = 1.45 years; see **Table 3** for group descriptive statistics). All participants had competed at a National level, and 21 participants had additionally competed at an International level. Participant range of junior ranking under the International Tennis Federation was 60 to 220. These athletes typically trained for two to three sessions per day and 5–6 days per week. Their daily training involved completion of 60–180 min of tennis development and 30–90 min of strength and conditioning. This sample of athletes each competed in 80 to 100 matches in the year of their participation in this research. This project was approved by the University of South Australia's Human Research Ethics Committee. Adult participants provided written informed consent while for adolescent participants, written informed consent was provided by their parent or guardian prior to participation.

### Mindful attention awareness scale–adolescent

The Mindful Attention Awareness Scale – Adolescent (MAAS-A; Brown et al., 2011) is a single-factor measure of dispositional mindfulness among adolescents. Mindfulness is defined in this scale as “a receptive state of attention that, informed by an awareness of present experience, simply observes what is taking place” (Brown et al., 2011, p. 1024). Each of the 14-items uses a six-point scale ranging from 1 *(Almost always)* to *6 (Almost never)*. The MAAS-A considers the absence of mindful attention in various situations (e.g., “*I find myself preoccupied with the future or the past”; “I snack without being aware that I'm eating*”) and is scored by calculating the average across all items, with higher scores reflecting greater dispositional mindfulness. Strong internal consistency and acceptable test-retest reliability of the MAAS-A has been established in adolescent populations (Brown et al., 2011). The MAAS-A was included to allow for comparison of dispositional mindfulness between groups as a potential covariate. In the present study, participant MAAS-A scores, which ranged between 2.43 and 5.64 (*M* = 3.88; *SD* = 0.75), were comparable to those reported in a previous study involving adolescent athletes (Chen and Meggs, 2020).

### Mindfulness meditation and control conditions

Participants in the FAM group were instructed to focus their attention on a single object (i.e., their breath), monitor for any distraction, and non-judgmentally redirect their attention back to the object in the case of any distraction. OMM participants were instructed to maintain awareness of all experiences (e.g., sounds, physical sensations, thoughts, self-talk) arising in the present moment. The control group listened to a recording unrelated to attention focusing or sport, involving an excerpt from a guide to garden maintenance (Rexford, 1915). This control listening task was employed as a control condition in previous research examining the influence of meditation on sequence learning (Chan et al., 2020). Each condition featured the same voice of an accredited, male meditation instructor and began with an identical 1 min 38 s section to introduce the exercise as an “attention focusing technique” and to instruct participants to adopt a comfortable, seated posture. Immediately after completing the mental exercise, participants took position on the tennis court to complete the serve return task. Participants were instructed to return each serve as effectively as possible while aiming for the target zone, with both speed and accuracy being equally important. Finally, prior to each task block, participants were given the following instructions relevant to their group:

FAM: “As you perform this tennis task, always use narrow, focused attention like you did in the attention technique.”

OMM: “As you perform this tennis task, always use expansive, all-inclusive attention like you did in the attention technique.”

Control: “As you perform this tennis task, always use your attention like you did in the attention technique.”

### Sequenced tennis task

**Figure 1:**
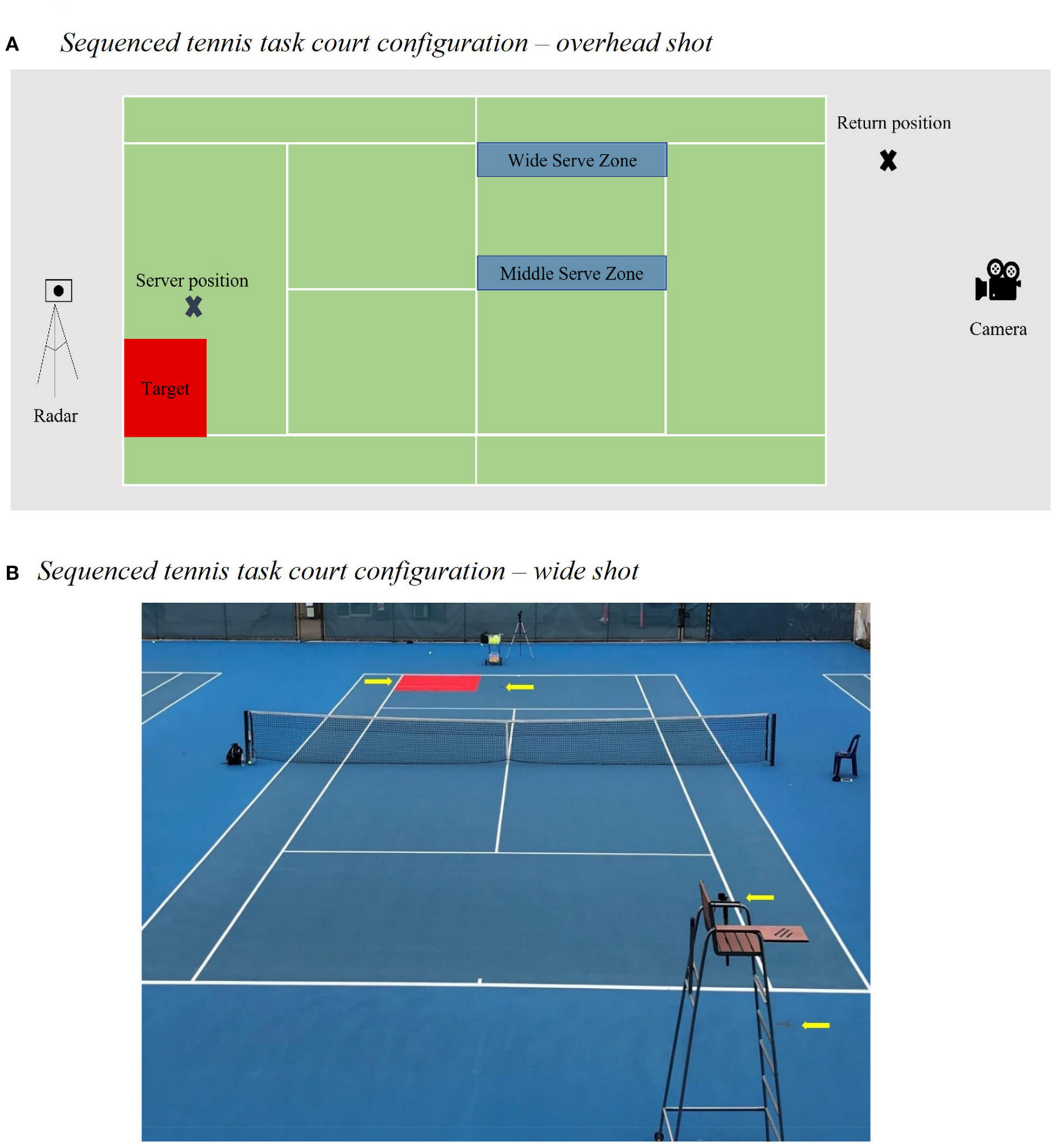
Tennis court configuration for implicitly sequenced serve return task.

The tennis serve-return task devised for this study included key performance elements from the SRTT paradigm (Nissen and Bullemer, 1987). Specifically, rather than key press responses to visual stimuli on a monitor, athletes were instructed to return serves such that the return landed on a 2,740 mm by 2,740 mm target space in the opposite sideline/baseline corner of the service court. Like the SRTT, athletes responded to one of four serve types, which unbeknownst to them followed a second-order conditional 12-serve sequence. Athletes completed this task on a competition standard tennis court, as illustrated in Figure 1.

Participants completed three blocks, each consisting of 24 serve return trials. Blocks 1 and 2 included two cycles of the 12- serve sequence. This sequence included four variations based on serve (flat/slider) and location (wide/middle), giving a second order conditional sequence featuring 3 repetitions of each serve variation (i.e., 121432413423; see Table 1 for serve variation details). Flat serves prioritize power and are the fastest serve type, whereas slide serves balance power and spin. Kick serves are the slowest serve type and involve the greatest amount of spin. Regarding serve location, “wide” serves were aimed close to the sideline of the service box, whereas “middle” or “T” serves were aimed at the center service line of the service boxes.

**Table 1:** Serve number, type, location, and velocity characteristics.

**Serve number**	**Serve**	**Location**	**Mean velocity (km/h) *(SD)***
1	Flat	Wide	134.96 (6.97)
2	Flat	Middle	140.04 (7.27)
3	Slide	Wide	123.55 (6.78)
4	Slide	Middle	128.25 (6.57)
5	Kick	Wide	112.11 (9.69)
6	Kick	Middle	113.48 (7.64)

To assess reliance on the trained sequence structure, the third block contained two cycles of a new 12-trial sequence, which included pre-learned serves as well as two new serves (kick wide/kick middle). This new sequence followed a different second order conditional pattern and included two repetitions of each variation (i.e., 353421646152). Alteration of the second order conditional sequence and inclusion of un-trained stimuli in block 3 allowed for investigation of the transfer of performance to new, untrained contexts. Like the SRTT, the extent to which performance in the final learning block was reliant on the underlying sequence (and thus the extent to which performance was plan- or stimulus-based) is inferred based on the magnitude of performance decline in the subsequent transfer block (see Chan et al., 2017). Performance declines from block 2 to block 3 of greater magnitude reflect plan-based responding, whereas smaller performance declines reflect stimulus-based responding. Across the task, trial duration was standardized at 15 s per trial.

A Tennis Australia qualified high-performance coach, blinded to participant condition, acted as the server, and followed the structured sequence. Serves were identified as valid if they landed in the regulation service area. Any serve that did not land in the service area, for example by missing wide, long, or by hitting the net, were categorized as fault serves. Serve location was standardized at a point 2.00 m inside the baseline and 3.50 m inside the left sideline. This location was chosen, rather than the standard serving position behind the baseline, to prioritize serve accuracy.

On each trial, participants were required to assume return-position at a standardized location 1.0 m behind the baseline, with the right foot in line with the right sideline. From this return location, participants were instructed to return each serve as accurately as possible whilst aiming for a 2.74 × 2.74 m target placed in the opposite baseline/side-line corner. Return outcomes were recorded for each trial following the definitions outlined in Table 2. For example, serves were initially classified as “returned” if the athlete contacted the ball after a single bounce. As such, all serves would be classified as ‘returned' unless the serve was an ace (i.e., the returner failed to make any contact with the ball). Returned serve outcome was the most basic level of analysis, with the other return outcomes nested within the previous level. For example, a target hit outcome would satisfy criteria as a returned serve outcome and an in-bounds return.

**Table 2:** Tennis task serve return outcomes.

**Return outcome**	**Definition**
Returned	Contact made with the ball after a single bounce
In-bounds	Ball is returned and lands inside the return court
Target hit	Ball is returned and lands on the target

A tripod-mounted radar gun (Stalker Pro 2, Applied Concepts, U.S.A) was positioned 3.50 m behind the baseline, in line with the serve position and at a height of 1.40 m to monitor serve velocity of each trial. The appropriate serve was cued by the primary investigator, who stood behind the radar gun and discreetly informed the server of the subsequent serve as they collected their next tennis ball. All Adelaide participants (*n* = 16) faced the same server (Server 1). In Sydney, 12 participants faced Server 2 and two participants faced Server 3. Participant mean return accuracy did not significantly differ between all three servers (*p* = 0.67).

The task was video recorded at a frame rate of 120 frames per second (Hero 5 Black, GoPro Inc. U.S.A) to allow for subsequent performance analyses. The camera was positioned 3.50 m behind the receiver's baseline and 1.40 m in from the right sideline. Camera height was standardized at 2.20 m. This positioning allowed for single-frame analysis of server and receiver and has been used in previous analyses of tennis serve return performance (e.g., Williams et al., 2002, 2004). Return scores were assessed and recorded during the task by the primary investigator. Video recordings were then consulted to confirm serve validity and return outcome of each trial.

### Procedure

Athletes completed an online questionnaire assessing basic demographic information and dispositional mindfulness (MAAS-A) approximately 1 week prior to testing. Following this, athletes were pseudo-randomly allocated into one of three experimental groups based on age (older or younger than 16.5 years) and gender (all participants reported either Male or Female). Groups were defined by the mental exercise completed prior to task performance and included focused attention meditation (FAM), open monitoring meditation (OMM) and control. Participants were blinded to their experimental condition until completion of data collection. The meditation techniques and control condition were referred to as being a “mental exercise” or “attention focusing technique” at all times to avoid any expectancy effects related to preconceived notions of concepts related to mindfulness or meditation.

**Figure 2:**
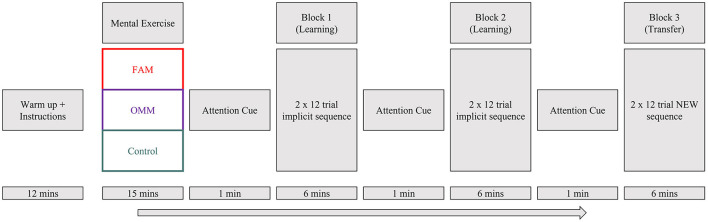
Experimental procedure. FAM, Focused attention meditation; OMM, Open monitoring meditation. Attention cues verbally reminded athletes to regulate attention in alignment with how they used their attention during the mental exercise.

As detailed in Figure 2, each participant completed a standardized 10-min warm up, before receiving initial instructions and completing the mental exercise relevant to their condition. The 15-min mental exercise guided the participant through a meditation technique or control listening task. Athletes wore headphones and an eye-mask to reduce distractions and were seated in a chair next to the court. Prior to the first task block participants were given the verbal attention-focusing cue relevant to their condition. These same instructions were repeated in the 1-min rest periods prior to blocks 2 and 3.

### Data analyses

All statistical analyses were conducted in *R Studio* (R Core Team, 2021). Participant gender, handedness, age, and dispositional mindfulness (MAAS-A) characteristics were analyzed for group differences based on Chi-Square and analysis of variance (ANOVA) as appropriate to the class of measure. The sequenced tennis task performance dataset included a total of 2,160 trials, 215 (9.95%) of which were faults (i.e., serves which landed outside of the regulation service box). To prepare data for analyses, fault serves were first examined to rule out systematic group by block differences. As no significant group differences (*p* = 0.06) or group by block interaction (*p* = 0.25), were observed for the occurrence of faults, these trials were removed from further analysis.

Subsequently, for each participant and tennis task block, the mean serve velocity of non-fault serves was calculated. Mean serve velocity was submitted to ANOVA to test for main effects and interactions of group, block, gender, and server factors.

For each participant and tennis task block, the odds ratio of returned serves was calculated based on a ratio of trials classified as a “returned” outcome and the total number of non-fault trials. Then, for all returned serve trials, the odds ratio of in-bounds returns was calculated for each participant and block based on the ratio of trials classified as an in-bounds return outcome and the total number of trials classified as a return outcome. Finally, for all in-bounds returns, the odds ratio of target hits was calculated for each participant and block based on the ratio of trials classified as a target hit outcome and the total number of trials classified as an in-bounds return outcome.

Odds ratios for serve returns, in returns and target placement were separately analyzed using beta regression modeling with the glmmTMB (Brooks et al., 2017) package. Models included group, block, gender, and serve velocity as fixed factors and participant as a random factor:


$$\begin{array}{l} Odds\;Returned\;Serve_{i}=\;\beta_{0}+\;\beta_{1}Group_{i}+\;\beta_{2}Block_{i}\\ +\;\beta_{3}Gender_{i}+\beta_{4}Serve\;Velocity_{i}+participant_{0i}+\;\varepsilon_{i}\\ Odds\;In-bounds\;return_{i}=\;\beta_{0}+\;\beta_{1}Group_{i}+\;\beta_{2}Block_{i}\\ +\;\beta_{3}Gender_{i}+\beta_{4}Serve\;Velocity_{i}+participant_{0i}+\;\varepsilon_{i}\\ Odds\;Target\;Hit\;Return_{i}=\;\beta_{0}+\;\beta_{1}Group_{i}+\;\beta_{2}Block_{i}\\ +\;\beta_{3}Gender_{i}+\beta_{4}Serve\;Velocity_{i}+participant_{0i}+\;\varepsilon_{i} \end{array}$$

Model fit was evaluated using diagnostics from the DHARMa (Hartig, 2020) package. For the three models, Kolmogorov-Smirnov, overdispersion and outlier tests and deviation between model residuals and predicted values were not significant. Type II Wald tests were conducted to assess significance of modeled fixed factors main effects and interactions. *Post-hoc* analysis of significant fixed factor main effects or interactions was conducted using pairwise comparison, with Tukey correction, of estimated marginal means using the emmeans (Lenth, 2021) package. Means and 95% confidence intervals from beta regression models are interpreted as the proportion of returned serves, in-bounds returns, or target hit returns.

## Results

### Participant and tennis task characteristics

No significant group differences were observed in gender distribution (*p* = 0.91), handedness (*p* = 0.24), age (*p* = 0.70), MAAS-A score (*p* = 0.53), or proportion of participants at each testing site (*p* = 0.98). Chi-square analyses revealed no significant differences in the proportion of trials by each server (*p* = 0.91). ANOVA analyses of serve velocity revealed a main effect of block [*F*_(2, 48)_ = 13.19, *p* < 0.001] and gender [*F*_(1, 48)_ = 4.74, *p* < 0.05] but no other significant main effects or interactions. The main effect of block was based on mean serve velocity in block 3 (125.78 km/h) being significantly slower than in block 1 (130.38 km/h, *p* < 0.01) and block 2 (132.06 km/h, *p* < 0.001). Serve velocity was not significantly different between block 1 and block 2 (*p* = 0.39). Serve velocity for females (128.04 km/h) was significantly slower than for males (130.76 km/h), though the magnitude of this difference was only 2.72 km/h. See Table 3 for descriptive statistics.

**Table 3:** Age, trait mindfulness, testing site, gender, handedness, and serve velocity in the meditation and control groups.

	**Group**		
	**FAM**	**OMM**	**Control**
Age Mean (SD)	16.30 (1.59)	16.13 (1.21)	16.70 (1.65)
MAAS-A Mean (SD)	4.09 (0.81)	3.75 (0.62)	3.79 (0.85)
Testing site Adelaide/Sydney %	54.55/45.45	54.55/45.45	50/50
Gender Male/Female %	54.55/45.45	45.45/54.55	50/50
Handedness Right/Left %	100/0	100/0	87.5/12.5
Serve velocity km/hr Mean (SD)	130.51 (5.08)	127.93 (4.86)	129.91 (5.81)
Serve velocity km/hr Min – Max	123.05–144.75	117.55–137.90	118.38–144.36

FAM, Focused attention meditation; MAAS-A, Mindful Awareness Attention Scale – Adolescent; OMM, Open monitoring meditation.

### Returned serve outcomes

Modeling of returned serve odds revealed mean serve velocity [*X(1)* = 10.64, *p* < 0.01], and block [*X(2)* = 21.33, *p* < 0.001] as significant model parameters. The block parameter was superseded by a significant group by block interaction term [*X(4)* = 10.93, *p* < 0.05]. No other fixed factors or interactions were significant. The mean serve velocity parameter reflected a 0.082 decrease in returned serve proportion for each unit increase in serve velocity. For the group by block interaction, pair-wise comparisons revealed no significant group differences at block 1 (all *p* > 0.98), 2 (all *p* > 0.37) or 3 (all *p* > 0.73). However, for the control group, proportion of returned serves was significantly higher at block 2 (0.96, 95%CI: 0.91, 0.98) than block 1 (0.87, 95%CI: 0.75, 0.93, *p* < 0.001) and block 3 (0.85, 95%CI: 0.721, 0.93, *p* < 0.01), while block 1 and 3 did not differ significantly (*p* = 1.0). Both FAM (all *p* > 0.06) and OMM (all *p* > 0.59) groups did not demonstrate significant differences in returned serve proportion across the three blocks. Group by block returned serve proportions are presented in **Figure 4A**.

### In-bounds return outcomes

**Figure 3:**
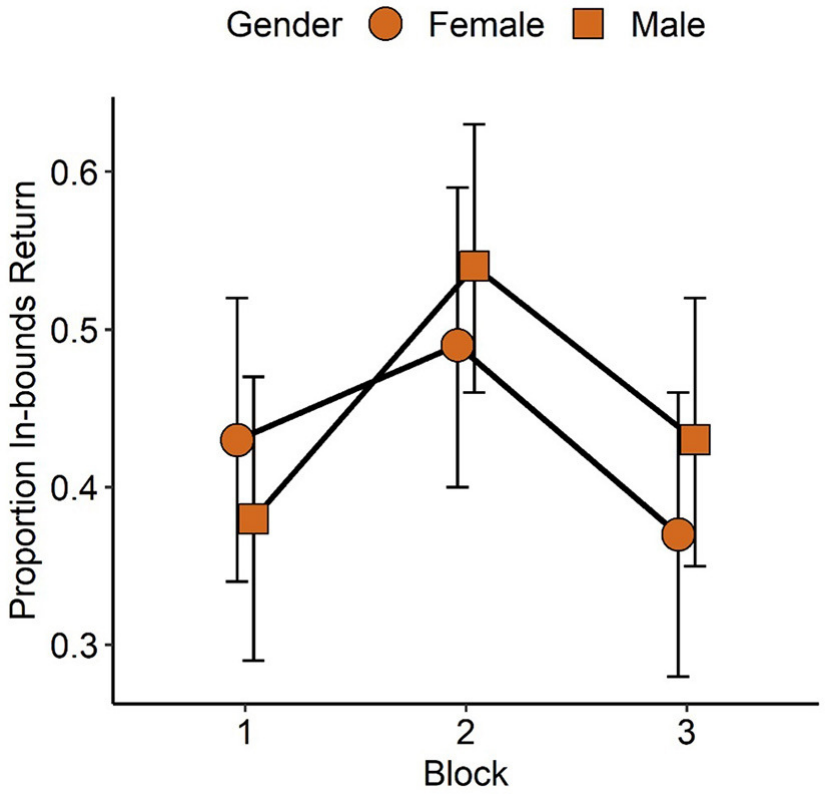
Proportion of serve returns that were placed in-bounds as a function of participant gender groups and sequenced tennis task blocks. A significant gender by block interaction (*p* < 0.05) was based on males exhibiting significant increase in proportion of in-bounds returns between block 1 and 2 but then no significant change to block 3, which was a transfer block involving novel serves and a novel serve sequence. Females did not exhibit significant changes between blocks 1 and 2 but then a significant decline in proportion of in-bounds returns at block 3. Error bars represent 95% confidence intervals.

**Figure 4:**
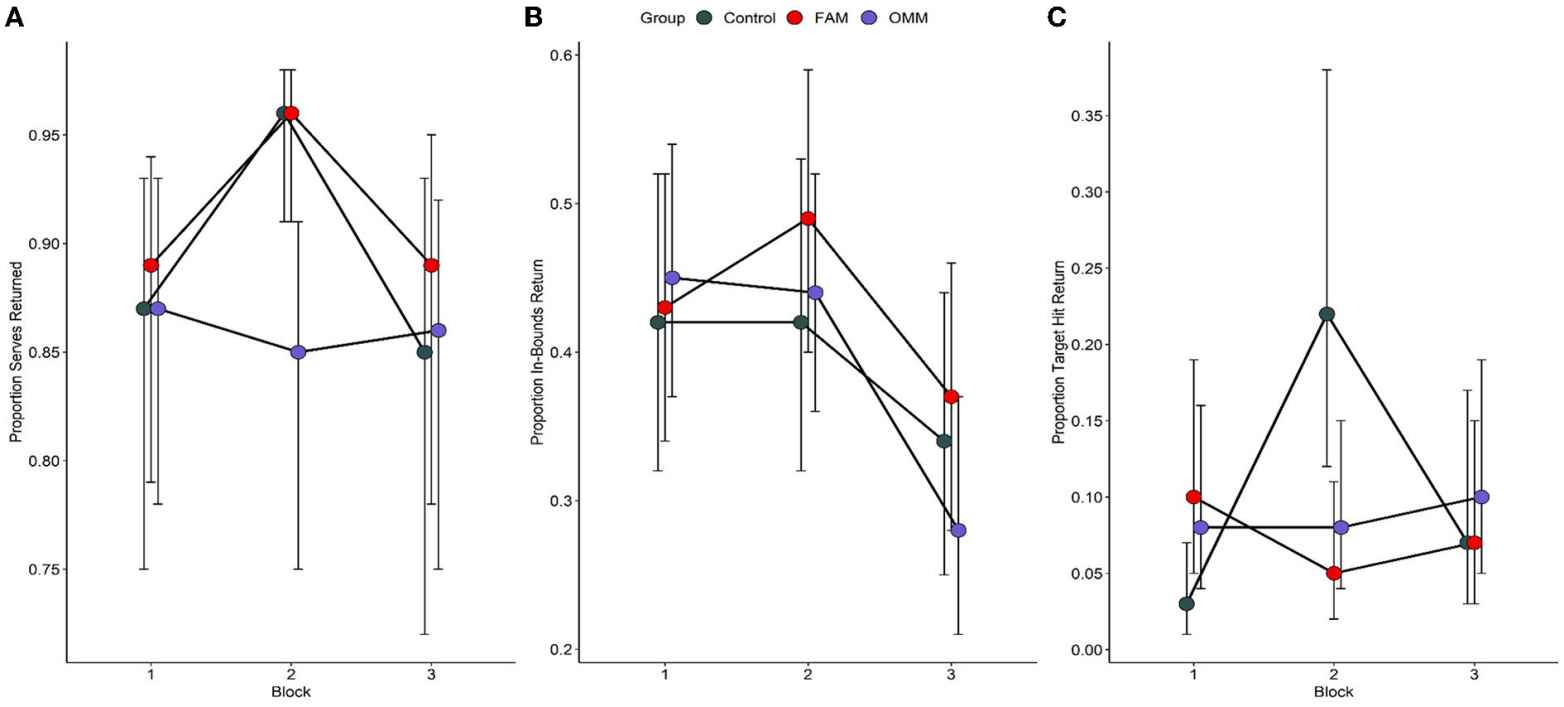
Proportion of returned serves **(A)**, returned serves placed in-bounds **(B)**, and target hits **(C)** as a function of mindfulness meditation or control groups and sequenced tennis task blocks. A significant group by block interaction was observed for the proportion of returned serves (*p* < 0.05) based on a significant increase in returned serve proportion between blocks 1 and 2 and then a significant decline in proportions at block 3 for the control group. No significant changes across blocks were observed for FAM and OMM groups. There was no significant group by block interaction for the proportion of in-bounds returns (*p* = 0.28). A significant group by block interaction was observed for the proportion of returned serves placed within a target area (*p* < 0.001) based on a significantly higher target hit proportion for the control group as compared to the FAM group in block 2. Furthermore, a significant increase in target hit proportion between blocks 1 and 2 and then a significant decline in proportions at block 3 for the control group while no significant changes across blocks were observed for FAM and OMM groups. In blocks 1 and 2 of the sequenced tennis task, four serves were sequenced in a repeating 12-serve second-order conditional sequence unbeknownst to the returner. In block 3, the sequence was altered by introducing two novels serves and presenting the serves in a novel 12-serve second-order conditional sequence. Prior to block 1 of the task, participants completed a brief single session of focused-attention meditation (FAM), open-monitoring meditation (OMM) or a control condition involving listening to an audio book. Error bars represent 95% confidence intervals.

Modeling of in-bounds return odds revealed mean serve velocity [*X(1)* = 10.08, *p* < 0.01], block [*X(2)* = 14.97, *p* < 0.001] and gender [*x(1)* = 4.79, *p* < 0.05] as significant model parameters. The block and gender parameters were superseded by a significant gender by block interaction term [*X(2)* = 6.68, *p* < 0.05]. No other fixed factors or interactions were significant. Based on the serve velocity parameter, proportion of in-bounds return decreased by 0.039 for each unit increase in serve velocity. In-bounds return proportion did not differ significantly between males and females in block 1 (*p* = 1.0), 2 (*p* = 0.41) or 3 (*p* = 0.068). Females exhibit higher in-bounds return proportion in block 2 (0.49, 95%CI: 0.40, 0.59) than block 3 (0.37, 95%CI: 0.28, 0.46, *p* < 0.05) but no significant difference compared to block 1 (0.43, 95%CI: 0.34, 0.52, *p* = 0.99), and blocks 1 and 3 did not differ significantly (*p* = 0.07). In contrast, males exhibit higher in return proportion in block 2 (0.54, 95%CI: 0.46, 0.63) than block 1 (0.38, 95%CI: 0.29, 0.47, *p* < 0.05) but no significant difference compared to block 3 (0.43, 95%CI: 0.35, 0.52, *p* = 0.24), and blocks 1 and 3 did not differ significantly (*p* = 0.99). Group by block in return proportions are presented in **Figure 4B**, and gender by block in return proportions are presented in Figure 3.

### Target hit return outcomes

Modeling of target hit return odds revealed a significant group by block interaction term [*X(4)* = 22.66, *p* < 0.001]. No other fixed factors or interactions were significant. Pair-wise comparisons revealed no significant group differences in target hit proportion in block 1 (all *p* > 0.25). In block 2, the control group (0.22, 95%CI: 0.12, 0.38) demonstrated significantly higher target hit proportion than the FAM group (0.05, 95%CI: 0.02, 0.11, *p* < 0.05) but no significant difference to the OMM group (0.08, 95%CI: 0.04, 0.15, *p* = 0.53). Furthermore, the target hit proportion did not significantly differ between FAM and OMM groups (*p* = 1.0) in block 2. There were no significant group differences in block 3 (all *p* = 1.0). For the control group, block 2 the proportion of target hits was significantly higher than block 1 (0.03, 95%CI: 0.01, 0.07, *p* < 0.01) but was not significantly different to block 3 (*p* = 0.36). Target hit proportion did not significantly differ across blocks for FAM (all *p* > 0.97) and OMM (all *p* > 0.85). Group by block target placement return proportions are presented in Figure 4C.

## Discussion

This study investigated the instantaneous effects of single-session meditation on tennis serve-return performance in elite, adolescent athletes. In line with previous laboratory research, it was hypothesized that both FAM and OMM meditation techniques would enhance subsequent task performance compared to a control condition. In addition, the extent to which performance relied on the trained sequence or features of the individual serve stimuli was compared between groups in a transfer block. These effects were assessed using an applied tennis task which required athletes to respond to blocks of tennis serves which followed an implicitly sequenced order. The present results suggest that the instantaneous benefits of FAM and OMM may not extend to complex motor tasks such as the tennis serve return. Moreover, compared to a control listening condition, meditation may have impaired acquisition of sequential information.

### Meditation-related performance enhancement

Results did not support the hypothesis that meditation would enhance serve return performance relative to a control listening condition. For example, analyses of “returned” serves, indicating whether the participant was able to make any contact between racquet and ball, suggested equivocal performance between FAM, OMM and control groups within each task block. Given that the alternative to a returned serve was failure to make any contact with the ball, this equivalency in returned serve proportions between groups suggests that meditation did not reduce the likelihood of being “aced.” Similarly, groups did not differ within any task block in the proportion of serves returned in-bounds, suggesting that meditation did not lend any significant benefits in terms of providing a return that would be deemed valid in a match-play context.

In contrast to returned serves and in-bounds outcomes, a significant group difference did emerge for target hit returns. However, contrary to hypotheses, this difference favored the control condition. Specifically, in block 2 the control condition recorded a significantly higher proportion of target hits relative to FAM. Why control participants outperformed FAM in this block is not immediately obvious, though the results suggest that meditation may have impaired performance gains across the two learning blocks. Whereas the control condition significantly improved from block 1 to block 2 in the proportion of both returned serves and target hits, neither FAM nor OMM displayed any change in serve return performance. Thus, the present data suggests that the completion of a single bout of either focused or open-monitoring meditation had almost no bearing on performance outcomes (i.e., accuracy of responding) in a subsequent, implicitly sequenced tennis serve return task. To the limited extent that any group differences did emerge, these were in favor of the control condition, who listened to an audiobook prior to the tennis task.

### Differential forms of sequential performance

It was also hypothesized that participants who completed OMM would utilize plan-based responding to a greater extent than FAM participants, due to a state of weakened cognitive control established during OMM. Conversely, a single bout of meditation which strengthened cognitive control (FAM) would result in greater stimulus-based responding and thus reduced sequence dependency. These differential forms of sequential performance were inspected by comparing performance in the final learning block (block 2) to a transfer block (block 3) in which the implicit sequence was altered. According to hypotheses, a significant performance detriment was expected between blocks 2 and 3 for OMM, reflecting plan-based responding, whereas FAM was expected to maintain performance into the transfer block due to greater reliance on stimulus-based responding.

Results did not support the hypothesis of differential sequential performance between meditation types, with both FAM and OMM exhibiting equivalent performance across all task blocks irrespective of the presence of a trained sequence. In contrast, the control condition exhibited significant variability in performance. After the initial task block, whereas meditation groups showed no performance changes, control participants improved significantly in the proportion of returned serves and target hits in the second task block. Controls also exhibited a significant decline in both returned serves and target hit proportions when the underlying sequence was altered in block 3. The performance detriment in the transfer block for the control group could suggest that controls, compared to meditation groups, acquired greater sequential information during the learning blocks, thus facilitating greater plan-based responding in block 2. Conversely, performance following meditation was maintained following changes to the underlying sequence, suggesting that meditation may have impaired acquisition of sequential information and/or prioritized stimulus-based responding.

Interestingly, whilst meditation techniques did not appear to elicit observable differences in learning, a gender difference did emerge for in-bounds return outcomes. Irrespective of experimental condition, male athletes significantly increased the proportion of in-bounds returns from the first to the last learning block. In addition, male athletes maintained their in-bounds return performance into the transfer block. Female athletes did not significantly increase the proportion of in-bounds returns across learning blocks, and in-bounds return performance suffered significantly when the implicit sequence was removed in the transfer block. Overall, this pattern of results could suggest that, compared to males, female athletes acquired greater sequential information and were thus more anticipatory in their return approach in the final learning block. However, this may not be a reliable interaction as gender was only a significant parameter in modeling of in-bounds return outcomes, and thus block by gender interactions were not observed for proportions of returned serves and target hits.

### General discussion

Overall, the present pattern of results is markedly different to previous laboratory research (Chan et al., 2017, 2018, 2020; Immink et al., 2017), in which single session meditation has been associated with augmented performance on subsequent implicitly sequenced tasks, with divergent forms of responding following FAM vs. OMM. Several potential explanations can be proffered to explain the divergence between current and previous findings. Firstly, it is possible that the meditation techniques may not have sufficiently manipulated participants' cognitive control states. Whilst the meditation and control techniques have previously been shown to effectively induce altered cognitive control states in laboratory settings in general population, meditation naïve adults (Chan et al., 2017, 2018; Immink et al., 2017), it is possible that the adolescent athletes did not sufficiently adhere to the attention regulatory instructions provided in the meditation techniques to derive cognitive control augmentation. No subjective or objective measures of meditation engagement were implemented, and thus it is difficult to determine the athletes' experiences whilst completing the meditation or control techniques. However, it is possible that participants in the meditation condition may have struggled to follow the technique and instead engaged in daydreaming or similar default mode network activity (Garrison et al., 2015). Along these lines, it should not be assumed that findings from adult populations necessarily generalize to younger populations (e.g., Friedman et al., 2009) whose cognitive control has yet to fully develop (Ferguson et al., 2021). It is possible that younger individuals who are naïve to meditation may not be able to establish and sustain meditation states to the same extent as adults. Future research is required to investigate potential benefits of single session meditation in relation to the developmental trajectory of cognitive control.

Perhaps the most parsimonious explanation for why meditation did not appear to enhance performance, nor influence plan- and stimulus-based responding, is that the present research investigated performance in an applied sporting setting with a complex, gross motor task (i.e., the tennis serve return). In contrast, previous laboratory research investigating meditation-related augmentation of performance utilized simple keyboard press tasks (Chan et al., 2017, 2018; Immink et al., 2017). Whereas laboratory tasks featuring simple skills allow for stringent experimental control, and highly precise measurement, the generalisability of these tasks to more complex skills has been repeatedly questioned (Wulf and Shea, 2002; Sternad et al., 2014; Levac et al., 2019). For example, Levac and colleagues (Levac et al., 2019) argue that complex real-world tasks, from brushing one's hair to returning a tennis serve, are significantly different to simple laboratory tasks (e.g., key pressing) because complex skills involve ‘nested redundancy' and thus can be achieved through a functionally infinite number of possible solutions. Whereas the keyboard press responses of the traditional SRTT can only be achieved by depressing certain keys with specific, predetermined fingers (i.e., low redundancy), the serve-return task implemented in the present study features a comparatively broad objective (i.e., return the ball whilst aiming for the target) that can be achieved through any one of limitless combinations of bodily movements, ball trajectories, and many other factors (i.e., high redundancy). Whilst evidence suggests that single session meditation may instantaneously bias performance in the simple laboratory tasks, presumably via altered cognitive control states (Chan et al., 2017, 2018; Immink et al., 2017), these meditation-related effects might not have been sufficient to exert observable influence on the complex, real-world task implemented in the present study. Put simply, it may be that the instantaneous effects of meditation on skilled performance do not extend to complex, sport-specific skills that involve greater perceptual and motor demands. However, this interpretation does not explain why the control condition exhibited a divergent pattern of performance compared to both FAM and OMM. If the effects of single-session meditation had no influence over complex skill performance then it would be logical to assume that patterns of serve return performance would have been equivalent following meditation or control. Further research is required to elucidate whether single-session meditation may instantaneously bias subsequent complex motor skill performance.

## Limitations

As an initial study that aimed to investigate the instantaneous effects of FAM and OMM on sequence learning in an applied sport setting, this study was subject to several limitations that must be considered when interpreting results. The tennis task was created to reflect key performance elements of the SRTT, a task which typically involves 12 learning blocks of 120 trials, giving 120 total cycles of the underlying sequence. However, due to practical limitations including time, as well as athlete and server fatigue, the tennis task only included four cycles of the trained sequence across blocks 1 and 2 (2 cycles per block), as well as 2 cycles of the transfer sequence in block 3. It is possible that this number of cycles may have been insufficient to allow appropriate formation of sequential structures. Additionally, whilst each was an expert, the human servers may not have provided the perfect stimulus for every trial. The Adelaide testing site was also outdoors and may have introduced greater variability through environmental conditions such as wind and sunlight. However, server and site were controlled through pseudo-random allocation procedures which reduces the likelihood of any systematic difference between groups. In addition, although this was the first translation of the SRTT to an applied setting, differential performance effects were observed between groups, suggesting that the task held sufficient sensitivity.

The generalizability of this research to tennis performance is also somewhat limited. For example, performance was operationalized only in relation to the landing spot of the serve return. This measures the accuracy of the return but does not capture the quality of the stroke. It is possible that some trials were poorly returned but happened to land in the service area or hit the target. It may be that athletes were able to return the ball, but no subjective measure of return quality was incorporated.

A final limitation is that the lead investigator, who was responsible for performance analyses, was not blinded to participant condition. As a result, bias was possible in the interpretation of serve returns which were difficult to score. However, this risk was significantly reduced by including follow-up video analyses. It is also possible that experimenter bias may have inadvertently exerted some influence over participant motivation. However, a scripted protocol was strictly adhered to throughout the study to minimize such bias.

## Conclusion

In a sample of elite, adolescent tennis athletes, instantaneous effects of meditation were investigated using an implicitly sequenced serve return task. The pattern of results in the present study was substantially different to those effects previously demonstrated in laboratory tasks. Neither FAM nor OMM was associated with improved performance relative to control, and meditation techniques did not appear to differentially influence the extent to which sequential performance reflected plan- or stimulus-based responding. It is possible that divergent findings between previous and current results may be attributable to participant characteristics (e.g., age, cognitive control development), task characteristics (e.g., greater complexity of the serve return skill), or perhaps a combination of both. Though emerging evidence suggests that single session meditation can instantaneously bias cognitive control states, further research is required to investigate whether these altered cognitive control states benefit performance in applied sporting contexts.

## Data availability statement

The original contributions presented in the study are included in the article/Supplementary material, further inquiries can be directed to the corresponding authors.

## Ethics statement

The studies involving human participants were reviewed and approved by University of South Australia, Human Research Ethics Committee. Adult participants provided written informed consent to participate. For participants under 18 years, written informed consent was acquired from both the participant and a legal guardian/next of kin prior to participation.

## Author contributions

EO'C contributed to the conception, design and formulation of the research questions and sequenced tennis task, conducted participant recruitment and data collection, contributed to data analyses, and wrote the initial manuscript. AM, MK, RC, and MI contributed to the conception, design and formulation of research questions and sequenced tennis task, provided ongoing oversight of the research, and revised the initial manuscript. MI additionally contributed to data analyses. All authors have read and approved the final version of the manuscript.

## Funding

EO'C was supported by an Australian Government Research Training Program Scholarship, and a Performance Sport Collaboration Scholarship from the Norwood Football Club.

## Conflict of interest

Author AM was employed by Tennis Australia. The remaining authors declare that the research was conducted in the absence of any commercial or financial relationships that could be construed as a potential conflict of interest

## Publisher's note

All claims expressed in this article are solely those of the authors and do not necessarily represent those of their affiliated organizations, or those of the publisher, the editors and the reviewers. Any product that may be evaluated in this article, or claim that may be made by its manufacturer, is not guaranteed or endorsed by the publisher.
